# Clinical Utility of Cognistat in Multiprofessional Team Evaluations of Patients with Cognitive Impairment in Swedish Primary Care

**DOI:** 10.1155/2014/649253

**Published:** 2014-03-23

**Authors:** Maria M. Johansson, Anna S. Kvitting, Ewa Wressle, Jan Marcusson

**Affiliations:** ^1^Department of Geriatric Medicine and Department of Clinical and Experimental Medicine, Linköping University, SE-581 85 Linköping, Sweden; ^2^Primary Health Care and Department of Medical and Health Sciences, Linköping University, SE-581 85 Linköping, Sweden

## Abstract

*Background*. Diagnostic evaluations of dementia are often performed in primary health care (PHC). Cognitive evaluation requires validated instruments. *Objective*. To investigate the diagnostic accuracy and clinical utility of Cognistat in a primary care population. *Methods*. Participants were recruited from 4 PHC centres; 52 had cognitive symptoms and 29 were presumed cognitively healthy. Participants were tested using the Mini-Mental State Examination (MMSE), the Clock Drawing Test (CDT), and Cognistat. Clinical diagnoses, based on independent neuropsychological examination and a medical consensus discussion in secondary care, were used as criteria for diagnostic accuracy analyses. *Results*. The sensitivity, specificity, positive predictive value, and negative predictive value were 0.85, 0.79, 0.85, and 0.79, respectively, for Cognistat; 0.59, 0.91, 0.90, and 0.61 for MMSE; 0.26, 0.88, 0.75, and 0.46 for CDT; 0.70, 0.79, 0.82, and 0.65 for MMSE and CDT combined. The area under the receiver operating characteristic curve was 0.82 for Cognistat, 0.75 for MMSE, 0.57 for CDT, and 0.74 for MMSE and CDT combined. *Conclusions*. The diagnostic accuracy and clinical utility of Cognistat was better than the other tests alone or combined. Cognistat is well adapted for cognitive evaluations in PHC and can help the general practitioner to decide which patients should be referred to secondary care.

## 1. Introduction

Assessments of potential cognitive impairment and diagnostic evaluations of dementia are often initiated and carried out in primary care. However, earlier studies have demonstrated that cognitive impairment remains undetected in many patients [1, 2]. It is important to identify cognitive impairment in primary care as soon as possible so that any reversible causes of cognitive decline can be identified. A correct dementia diagnosis is important so that good social support and medical care can be provided for patients and their relatives. Cognitive complaints by patients or questions about memory problems are less reliable sources of information than objective tests for detecting early cognitive impairment; therefore, sensitive cognitive screening tools are needed in primary care settings [3]. By combining a memory test with tests of executive function, orientation, attention, and judgement, care providers can better discriminate between healthy elderly and people with mild cognitive impairment (MCI) [4]. The most commonly used cognitive instrument for dementia evaluations is the Mini-Mental State Examination (MMSE) [5, 6], although it has many well-known limitations. The test results are influenced by age, education, socioeconomic background, and premorbid intelligence [7–9]. Swedish National Guidelines recommend using the MMSE together with the Clock Drawing Test (CDT) in the basic evaluation of dementia [10].

Other than the MMSE and CDT, few studies have investigated the efficacy of tests that can evaluate dementia in a primary care setting. In some primary care clinical settings in Sweden, occupational therapists (OT) use Cognistat (formerly known as the Neurobehavioral Cognitive Status Examination), a multidomain cognitive test to evaluate cognitive impairment. However, few studies have evaluated the validity and clinical utility of Cognistat for diagnosing dementia. Cognistat assesses several cognitive domains separately but does not sum global cognitive function [11, 12]. The test has been validated in a secondary care (SC) population by retrospectively comparing test results with final clinical diagnoses [13]. However, there are no data on the accuracy of the test for diagnosing dementia in a primary care setting in Sweden. This study investigated the diagnostic accuracy and clinical utility of Cognistat for identifying individuals with cognitive impairment in a primary care population. In addition, this study investigated the diagnostic accuracy of Cognistat compared with MMSE and CDT.

## 2. Materials and Methods

### 2.1. Design and Study Sample

The study has a cross-sectional design. Data were collected over 19 months, between 2007 and 2009, from 4 primary health care (PHC) centres in Linköping, a city in southeast Sweden. The community has around 150 000 inhabitants and the 4 PHC centres serve a population of 49 800 people; 11 200 of these residents were 65 years or older. The 4 PHC centres are socioeconomically characteristic of primary care in Linköping. There is a slightly older demographic profile in the population served by the 4 PHC centres; 22% of the population was over the age of 65 years compared with 16% in the overall primary care population in Linköping. The participants were systematically recruited from the 4 PHC centres during the selection period. All participants were asked to take part in the study during an appointment with a general practitioner (GP) and those who agreed provided written informed consent. The inclusion criteria were as follows: older than 65 years, any complaint or suspicion of cognitive symptoms expressed either by the patient, an informant, or primary care staff. In all, 52 people met these criteria. During the same period, patients visiting the GP for medical reasons other than possible cognitive symptoms were asked to participate in a clinical comparison group. Those participants extended the study group to assure its clinical relevance. Inclusion criteria for the comparison group were as follows: age older than 65 years and no complaint or suspicion of cognitive symptoms expressed either by the patient, an informant, or a GP. Twenty-nine people met the criteria and were willing to participate. These 29 participants were asked to complete a short questionnaire regarding self-estimated memory function and diseases/trauma related to the brain or head (Appendix). Exclusion criteria for all participants were a medical record of recent stroke, brain tumour, brain-related infection, head trauma, ongoing verified psychiatric illness, a previous dementia investigation, or a known dementia diagnosis. A study population of 81 patients were selected to provide an unsorted mixed primary care population with some suspicion of cognitive decline.

### 2.2. Instruments

Cognistat includes 10 subtests: orientation, attention, language (comprehension, repetition, and naming), constructional ability, memory, calculation, and reasoning (similarities and judgement) [3]. The language section also contains a subtest of qualitative aspects of word fluency, but this section is not scored. Each subtest, with the exception of memory and orientation, has a screening test. If the patient fails the screening item, a metric section is administered. A higher score indicates a higher level of function in each domain. Each subtest has a different scoring system (orientation 0–12; attention 0–8; comprehension 0–6; repetition 0–12; naming 0–8; constructional ability 0–6; memory 0–12; calculations 0–4; similarities 0–8; and judgement 0–6). The result is presented graphically and contains information about the level of impairment (normal/average, mild, moderate, and severe impairment). The test results are not presented as a global sum. Passing the screening test is considered normal/average. Cognistat has age-corrected norms and takes about 20 minutes to administer. In this study, Cognistat was administered in accordance with standardized instructions provided in the Swedish manual [11]. The cutoff point for impairment used in each subtest of Cognistat is included in Table 4 in the results section. These cutoff points include adjusted norms for construction, memory, and reasoning for people >65 years old. When analysing against diagnoses, a cutoff required one or more subtests to be in the impaired range for the participant to be considered cognitively impaired. Analyses were also made using a cutoff of two or more subtests within the impaired range as comparison.

The MMSE, which takes about 10 minutes to administer, assesses orientation, attention, memory, language, and visual construction [5]. The maximum score for the MMSE is 30 points. A cutoff of ≤26 points is used for cognitive impairment in the analyses. Analyses were also made using a cutoff of ≤23 points for comparison. These cutoffs have been used and recommended in previous studies [14, 15].

The CDT, which takes about 5 minutes to administer, is a cognitive instrument that measures visuospatial and executive functions [16]. Different versions and scoring methods exist. In this study, the CDT was administered by asking the participants to perform the following task on a blank sheet of paper: “Draw the face of a clock, put the numbers in the right place, and set the time to 10 past 11.” A five-point scoring scale was used whereby a perfectly drawn clock scored 5 points [16]. A minor visuospatial error scored 4 points. If an inaccurate representation of “10 past 11” is drawn but the visuospatial organization is well done, the score is 3 points. If the visuospatial disorganization of numbers is moderate, the score is 2 points. If the visuospatial disorganization is severe, the score is 1 point. If there is no reasonable representation of a clock, the score is 0. A cutoff of 4 or less is used for cognitive impairment in the analyses.

### 2.3. Procedures

**Figure 1:**
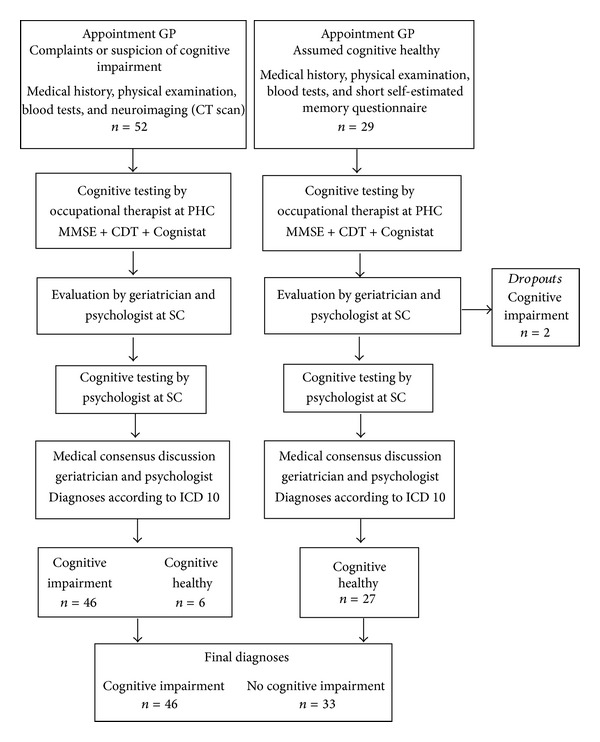
Flowchart of the diagnostic evaluation process. CDT, Clock Drawing Test; CT, computed tomography; GP, general practitioner; MMSE, Mini-Mental State Examination; PHC, primary health care; SC, secondary care.

The diagnostic evaluation process is presented in Figure 1. After the first visit with the GP, all 81 participants underwent the same cognitive testing procedure. The first appointment was with an OT at the patient's PHC centre where the cognitive tests were administered together with A Quick Test of Cognitive Speed (AQT) [17]. The AQT is analysed and presented elsewhere [18]. All the OTs who performed the tests had good experience and knowledge of the tests and used them routinely in their clinical practice. The results of these tests were not given to the psychologist or the geriatrician in their diagnostic work. Two to four weeks after seeing an OT, a psychologist and a geriatrician at a specialist geriatric unit (SC) evaluated all the participants. The tests used by the psychologist included the Alzheimer Disease Assessment Scale-Cognitive Subscale (ADAS-Cog) [19], Babcock Story Recall test (immediate and delayed recall) [20], the Letter Fluency test of the Delis-Kaplan Executive Function System (D-KEFS) [21], the Vocabulary Test of the Wechsler Adult Intelligence Scale-III (WAIS-III) [22], and the Trail Making Test A and B [23]. The tests used by the psychologist were chosen to cover a wide range of important cognitive domains such as general intellectual ability, attention, verbal and performance ability, episodic and semantic memory, and executive functions. These tests were part of the diagnostic workup.

### 2.4. Diagnosis of Cognitive Impairment

Diagnoses were made by consensus using the psychologists' and the geriatricians' examinations independent of the results from the cognitive test under study (i.e., they were blinded to these results). Diagnoses were based on the patient's case report, the patient's physical examination (including a neurologic examination), blood tests, neuroimaging, and the psychologist's assessment. The blood tests were done to exclude somatic causes for cognitive dysfunction. Etiologic diagnoses for dementia were initially set according to The ICD-10 International Classification (ICD-10) [24]. These etiologic diagnoses were validated and confirmed according to research criteria for probable Alzheimer disease [25], probable vascular dementia [26], Lewy body dementia [27], and Parkinson disease with dementia [28]. Patients with mixed dementia met the research criteria for probable Alzheimer disease but also had signs of ischemia on the computed tomography scan. These signs could not be directly linked to the cognitive symptoms. MCI was diagnosed according to criteria presented at a key symposium [29], which means that the patient was neither normal nor demented (according to Diagnostic and Statistical Manual of Mental Disorders, Fourth Edition (DSM-IV), or ICD-10 criteria) and there was evidence of cognitive decline (self- and/or informant reported and objectively measured cognitive decline over time). In addition, activities of daily living were preserved or complex instrumental functions were minimally impaired. The DSM-IV criteria were used to determine dementia. These criteria require the presence of both memory impairment and impairment in at least one additional cognitive domain and the presence of a cognitive impairment that interferes with social function or activities of daily living [30]. Cognitive impairment refers to any objective impairment of cognitive function according to the psychological evaluation.

### 2.5. Data Analysis

Quantitative analysis of the data was performed using SPSS for Windows 19.0 (SPSS, Inc., Chicago, IL). The chi-square and Fisher exact tests were used to compare differences in gender, native language, medical history, and medical drugs between the groups. Age, education, duration of symptoms, and test scores for MMSE, CDT, and Cognistat, and the neuropsychologist test battery were compared using the *t*-test. *P* values <0.05 were considered statistically significant throughout the analysis. Sensitivity, specificity, positive predictive value (PPV), and negative predictive value (NPV), and the area under the receiver operating characteristic (ROC) curve (AUC) with 95% confidence intervals were calculated using the final diagnoses as the standard. Clinical Utility Index (CUI+) ((sensitivity × PPV) − 1) according to Mitchell [31] and the Youden index (*J*) ((sensitivity + specificity) − 1) were calculated [32]. Analysis of the MMSE and the CDT combined was also done (the results of the two tests were analysed as one test and if participants scored under the cutoff in at least one of the tests, they were considered positive). The AUC was also analysed for each subtest of Cognistat.

## 3. Results

### 3.1. Final Diagnoses and Test Results

Among the group who visited primary care for reasons other than cognitive symptoms (*n* = 29), two participants had a medical history and obvious clinical signs that indicated undiagnosed cognitive decline when evaluated by the OT at the PHC and verified at the SC evaluation. These 2 participants were considered dropouts from the cognitively healthy group and their results were not analysed as part of the study. Of the 52 participants who visited a primary care facility primarily for suspected cognitive impairment, 6 were diagnosed as cognitively healthy. Thus, after the consensus discussion and SC diagnosis, 46 participants exhibited cognitive impairment and 33 participants did not (Figure 1). Of the 46 participants with a final diagnosis of cognitive impairment based on the criteria used in this study, 16 had MCI (35%), 12 had Alzheimer disease (26%), 5 had vascular dementia (11%), 6 had mixed dementia (13%), 2 had unspecified dementia (4%), 1 had Levy body dementia (2%), 1 had dementia from Parkinson disease (2%), and 3 had a comorbidity with depressive disorders (7%). Descriptive data and the test results for the study group after the final diagnoses are presented in Tables 1 and 2. The test results were significantly lower in the group with cognitive impairment (Table 2). The most prominent scores for the Cognistat subtests were found for memory and construction.

### 3.2. Diagnostic Accuracy of the Tests

**Figure 2:**
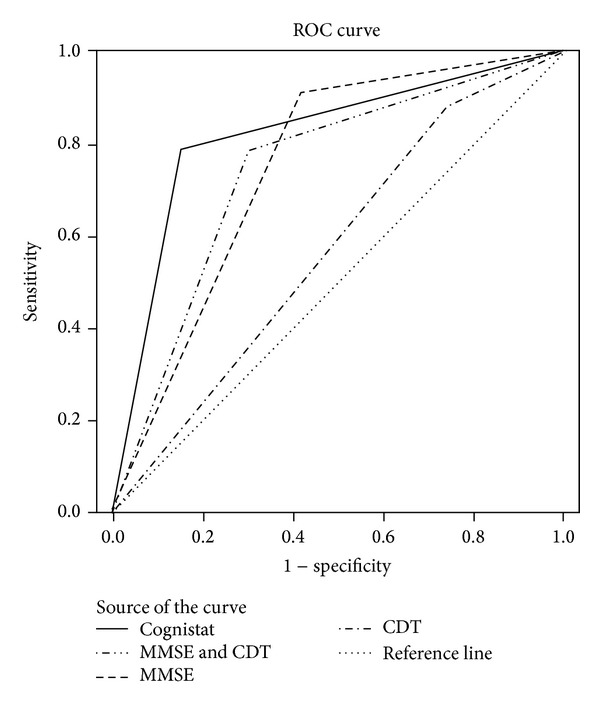
The ROC curves for Cognistat ≥1 subtest, MMSE ≤ 26, CDT ≤ 4, and combined MMSE and CDT ≤26 or ≤4. The AUC values are presented in Table 3. AUC, area under the ROC curve, CDT, Clock Drawing Test; MMSE, Mini-Mental State Examination; ROC, receiver operating characteristic.

The sensitivity, specificity, PPV, NPV, AUC, CUI+, and the Youden Index are presented in Table 3. The Cognistat had relatively good diagnostic accuracy with the best sensitivity of the three tests using a cutoff of at least one impaired subtest. However, the specificity and PPV for Cognistat were slightly lower than for the MMSE (cutoff ≤ 26), although they seem acceptable. When using the cutoff of at least two subtests within the range of impairment, Cognistat's specificity and PPV increased to 0.97 and 0.96, respectively, but the sensitivity decreased to 0.52. When comparing Cognistat with MMSE and CDT combined, Cognistat shows better results than combined tests (Table 3). With a Clinical Utility Index (CUI+) of 0.72, Cognistat was classified as good according to Mitchell [31], whereas the MMSE was satisfactory or moderate. This classification remains the same when the CDT is added. The CUI+ of the CDT was poor (0.20). ROC curves presenting the tests with highest cutoff are presented in Figure 2.

The sensitivity, specificity, and the AUC for each subtest of Cognistat are shown in Table 4. Memory and construction are the most prominent findings with an AUC of 0.84 and 0.73, respectively. The AUCs for other subtests of Cognistat were less than 0.70.

## 4. Discussion

This study found that Cognistat has relatively good sensitivity and PPV for detecting cognitive impairment and an acceptable specificity and NPV in a primary care population. The high sensitivity in combination with acceptable specificity and the high PPV indicates that the test is useful in primary care. The negative aspect of a test with high sensitivity and low specificity is the risk of overdiagnosis. On the other hand, tests with low sensitivity and high specificity increase the risk of underdiagnosis, with the added risk that patients and relatives do not receive adequate social support and medical treatment. This study investigated a population with a high proportion of patients with cognitive impairment; therefore the predictive values should be interpreted with this in mind. To evaluate the test in the whole primary care population, it would have been necessary to include all patients over the age of 65 years at the time of inclusion, a condition that was not feasible. On the other hand, we do not recommend screening all patients over the age of 65 years for cognitive impairment; this study investigated the clinical utility of Cognistat in primary care patients in whom some sort of cognitive decline was suspected.

The results indicate that Cognistat is more sensitive than the MMSE and yet still specific. Earlier research [13, 33, 34] supports our finding that the sensitivity of Cognistat was higher than that of the MMSE, but the relatively high specificity is more prominent in this study group than in earlier research, a difference that suggests that Cognistat would be useful in primary care settings. In comparison with MMSE and CDT combined, Cognistat is more sensitive with the same specificity and a slightly higher PPV. However, it is notable that the different subtests each have low sensitivity and the two subtests of memory and construction were the only subtests with an AUC >0.70. These two domains seem to be the most sensitive. The MMSE and CDT combined were the second best. Our results show that the MMSE and the CDT are not the ideal tests for detection of cognitive impairment. The study confirms earlier research suggesting that the MMSE is better at ruling out dementia than detecting it [5]. The CDT used alone seems to be a very insensitive instrument for detecting cognitive impairment. The MMSE and CDT are recommended in the national guidelines but our results indicate that there is a need for better cognitive instruments and that it is important to continue evaluating the tests in use today. One improvement is the ongoing work on a standardized Swedish version of the MMSE (MMSE-SR).

Cognistat is appealing as a cognitive instrument for primary care because it includes several cognitive subtests but is still easily administered. The multidomain perspective adds valuable information in a clinically applicable way with the possibility of presenting the results graphically and providing information about the level of impairment. DSM-IV requires memory and another cognitive domain to be impaired for a diagnosis of dementia and a test that includes more cognitive domains than memory might help the GP to determine a diagnosis. The screening and metric procedure in Cognistat is appealing and some qualitative data are provided during the test that are not presented in these figures. However, the test might be improved and even more sensitive with the addition of a short subtest of executive function.

The brief time it takes to perform the tests is important, especially in a clinical primary care setting. The Cognistat takes more time to administer than the MMSE, in some cases twice as long, but compared with other multidomain instruments, 20 minutes is acceptable. The economic cost must also be considered. The Cognistat has to be purchased unlike the MMSE and the CDT, which are free of charge. The extra time it takes to perform Cognistat has to be considered as a limited cost, if the test can lead to an early correct diagnosis and treatment and the benefit of lower social cost. In Sweden, the use of a multiprofessional team is recommended in the investigation and care of people with dementia [10]. It is not possible for the GP to administer more than a short test at the first appointment but other team members such as an OT or a nurse can administer other tests during another appointment and then discuss the results with the GP. It is notable that the GPs in the study had good clinical knowledge about their patients at the first primary care appointment. They erred on two of the healthy participants and, after the final diagnosis, 6 of the individuals with suspected cognitive decline. This strengthens the importance of doctor-patient continuity, a good medical history, and basic questions about cognitive function in primary care for early detection of cognitive decline. This area of clinical evaluation in the primary care setting needs to be further examined with regard to cognitive medical problems. The populations served by the 4 PHC centres included in this study had a slightly older demographic profile than the overall population, which might have contributed to the knowledge and interest in dementia evaluations among the GPs working there.

Some limitations need to be discussed. In this study, we used the cutoffs suggested in the Cognistat manual. As a consequence, participants with a high premorbid function could produce false-negative results due to a ceiling effect. A further limitation is the relatively small sample size. A larger study would be of interest to confirm the result. However, a power calculation indicated that a sample size of about 30 patients per group was sufficient. Our study does not analyse different subgroups of cognitive impairment. Van Gorp et al. [15] indicated that Cognistat did not help to differentiate between Alzheimer disease and vascular dementia. This remains to be investigated further with a larger sample. Another limitation is that the cognitively impaired group had a higher mean age and lower education level than the group with no cognitive impairment. These differences were not very large, but they are statistically significant. Although these characteristics may influence the test results, we used the geriatrician's and psychologist's evaluations as the standard. According to this evaluation, the participants with no impairment were cognitively healthy and were all evaluated in light of their premorbid function. By asking people who were visiting primary care facilities for reasons other than cognitive complaints to take part in the cognitively healthy group, we were trying to represent clinical reality; however, two participants were not cognitively healthy. This might have been avoided if an objective test was used to identify the cognitively healthy before inclusion or if a different approach was used to recruit the healthy comparison group. The group with cognitive impairment had a higher proportion of participants on salicylic acid, lipid-lowering drugs, and antihypertensive drugs. These are common vascular preventive medicines and therefore it is not surprising that their use was slightly higher in the group with cognitive impairment. However, the medical history did not differ significantly between the two groups.

The study results indicate that Cognistat, instead of or as a complement to the MMSE and the CDT, can be used in primary care as an initial instrument to evaluate cognitive impairment. A disadvantage of Cognistat might be that it does not include tests that measure processing speed or more demanding frontal or executive functions (some subtests, however, do incorporate measures of minor executive functions). Earlier studies have shown that this is an early symptom of dementia [35]. It may be profitable for future research to compare Cognistat with newer tests (e.g., the Cognitive Assessment Battery (CAB) [36] and the Montreal Cognitive Assessment (MoCA) [37] to detect MCI and early-stage dementia. These tests include more demanding or sensitive tests for executive functions and processing speed. This remains to be investigated further.

In Sweden, Cognistat is mostly administered by OTs. In addition to evaluating cognitive function, the OT can estimate the consequences of impairment in daily living and plan for further interventions that aim to restore or improve the functioning and activities of patients with cognitive impairment. The results of the test always need to be interpreted in the patient's daily life situation. None of the cognitive tests used in this study can be used alone to diagnose cognitive impairment; that is, the neuropsychology tests always need to be combined with other parts of the clinical evaluation, such as a medical history, diagnostic radiology, and a physical examination. In clinical use, a GP should consider the test results of each individual and interpret these within the overall status of the patient.

## 5. Conclusion

Cognistat shows fairly good diagnostic accuracy and clinical utility for detecting cognitive impairment in this primary care population. Memory and construction were the most prominent findings among the subtests included in Cognistat. Cognistat should be used for basic primary care evaluations to detect cognitive impairment overall, but it could also complement dementia evaluations in primary care facilities when the MMSE and CDT results are questionable. In addition, Cognistat can help a GP to determine which patients need to be referred to SC for further evaluation.

## Figures and Tables

**Table 1:** Descriptive data (mean ± standard deviation (SD)) for the cognitive impairment group and the no cognitive impairment group after the final diagnoses.

Variables	Cognitive impairment (*n* = 46)	No cognitive impairment (*n* = 33)	*P* value
Females/males	22/24	25/8	0.013
Age, years ± SD	79 ± 5.2	75 ± 5.5	0.004
Education, years ± SD	9.7 ± 3.6	11.5 ± 3.9	0.037
Native language, *n* (%)			
Swedish	42 (91)	33 (100)	0.136*
Non-Swedish	4 (9)	0 (0)	
Duration of cognitive symptoms, years ± SD	2.4 ± 1.9	0.3 ± 0.8	<0.001
Range	1–9	0–4	
Medical history, *n* (%)			
Cerebrovascular disease	5 (11)	1 (3)	0.392*
Ischemic heart disease	15 (33)	5 (15)	0.078
Hypertension	30 (65)	17 (52)	0.221
Diabetic disease	7 (15)	1 (3)	0.130*
Anxiety	4 (9)	0 (0)	0.136*
Mild depression	6 (13)	1 (3)	0.229*
Medical drugs, *n* (%)			
Warfarin	7 (15)	4 (12)	0.754*
Salicylic acid (low dose)	25 (54)	10 (30)	0.034
Lipid-lowering drug	25 (54)	10 (30)	0.034
Antihypertensive	38 (83)	18 (54)	0.007
Insulin	4 (9)	0 (0)	0.136*
Antidiabetic	3 (7)	1 (3)	0.636*
Antidepressant	18 (39)	7 (21)	0.091
Sleeping drug	16 (35)	5 (15)	0.051
Antipsychotic	4 (9)	0 (0)	0.136*
Anxiolytic	11 (24)	4 (12)	0.188

*P* value analysed by *t*-test for age, education, and duration of symptoms. Chi-square and Fisher exact test* for native language, medical history, and medical drugs. Significant *P* value <0.05.

**Table 2:** Test results (mean ± standard deviation) for the cognitive impairment group and the no cognitive impairment group after final diagnoses: confidence intervals of the mean difference and *P* values between the two groups.

Test	Cognitive impairment (*n* = 46)	No cognitive impairment (*n* = 33)	95% confidence interval	*P* value
MMSE, mean score ± SD	24.9 ± 3.7	28.6 ± 1.4	4.890–2.513	<0.001
CDT, mean score ± SD	4.5 ± 1.0	4.9 ± 0.4	0.693–0.004	0.048
Cognistat subtest (test range), mean score ± SD				
Orientation (0–12)	11.1 ± 1.7	11.9 ± 0.3	1.328–0.317	0.002
Attention (0–8)	5.7 ± 1.9	6.8 ± 1.4	1.784–0.296	0.007
Comprehension (0–6)	5.4 ± 0.9	5.9 ± 0.3	0.769–0.223	0.001
Repetition (0–12)	11.2 ± 1.4	11.9 ± 0.5	1.133–0.216	0.005
Naming (0–8)	7.4 ± 1.2	7.9 ± 0.3	0.872–0.121	0.011
Construction (0–6)	3.2 ± 1.7	4.5 ± 0.8	1.824–0.667	<0.001
Memory (0–12)	5.6 ± 2.9	9.5 ± 2.4	5.113–2.743	<0.001
Calculations (0–4)	3.6 ± 0.8	3.9 ± 0.3	0.610–0.078	0.012
Similarities (0–8)	5.8 ± 1.6	6.2 ± 0.7	1.398–0.340	0.002
Judgement (0–6)	4.6 ± 1.4	5.3 ± 1.0	1.227–0.145	0.014
Neuropsychology test battery, mean score ± SD				
ADAS-cog	13.41 ± 5.6	5.4 ± 2.6	6.092–9.886	<0.001
Babcock Story Recall test-immediate	3.57 ± 2.3	6.58 ± 2.7	4.191–1.830	<0.001
Babcock Story Recall test-delayed	3.44 ± 3.5*	9.42 ± 3.3	7.545–4.414	<0.001
Letter fluency test (D-KEFS), raw score	25.2 ± 11*	40.06 ± 11.5	19.993–9.639	<0.001
Letter fluency test (D-KEFS), scale score	7.53 ± 3.4*	11.7 ± 3.5	5.765–2.622	<0.001
The Vocabulary Test (WAIS-III), raw score	35.44 ± 13.4*	44.73 ± 8.1	14.158–4.408	<0.001
The Vocabulary Test (WAIS-III), scale score	9.47 ± 2.9*	11.39 ± 2.1	3.064–0.791	0.001
Trail Making Test A, seconds	99.5 ± 55.8**	44.06 ± 16.5^†^	37.581–73.294	<0.001
Trail Making Test B, seconds	252.7 ± 122.3***	136.38 ± 83.3^†^	63.321–169.413	<0.001

ADAS-cog, Alzheimer Disease Assessment Scale-cognitive subscale; CDT, Clock Drawing Test; D-KEFS, Delis-Kaplan Executive Function System; MMSE, Mini-Mental State Examination; SD, standard deviation; WAIS-III, Wechsler Adult Intelligence Scale-III. *t*-test used for all the test variables. Significant *P* value <0.05.

*n = 45.

**n = 44.

***n = 31.

^†^ *n* = 32 (due to internal dropouts).

**Table 3:** Diagnostic accuracy of the tests.

Test cutoff	Sensitivity (95% CI)	Specificity (95% CI)	PPV (95% CI)	NPV (95% CI)	AUC (95% CI)	*P* value (AUC)	CUI+	Youden Index (*J*)
Cognistat ≥1 subtest	0.85 (0.75–0.92)	0.79 (0.65–0.88)	0.85 (0.75–0.92)	0.79 (0.65–0.88)	0.82 (0.72–0.92)	<0.001	0.72 (good)	0.64 (0.41–0.78)
Cognistat ≥2 subtests	0.57 (0.48–0.59)	0.97 (0.85–0.99)	0.96 (0.82–1.00)	0.62 (0.54–0.63)	0.77 (0.66–0.87)	<0.001	0.55 (satisfactory)	0.54 (0.33–0.58)
MMSE ≤26	0.59 (0.49–0.63)	0.91 (0.78–0.98)	0.90 (0.76–0.97)	0.61 (0.52–0.66)	0.75 (0.64–0.86)	<0.001	0.53 (satisfactory)	0.50 (0.27–0.61)
MMSE ≤23	0.26 (0.19–0.26)	1.00 (0.90–1.00)	1.00 (0.72–1.00)	0.49 (0.44–0.49)	0.63 (0.51–0.75)	<0.049	0.26 (poor)	0.26 (0.09–0.26)
CDT ≤4	0.26 (0.17–0.32)	0.88 (0.76–0.96)	0.75 (0.50–0.91)	0.46 (0.40–0.50)	0.57 (0.44–0.70)	<0.292	0.20 (poor)	0.14 (−0.07–0.28)
MMSE + CDT ≤26 and ≤4	0.70 (0.60–0.77)	0.79 (0.65–0.89)	0.82 (0.70–0.91)	0.65 (0.53–0.74)	0.74 (0.63–0.85)	<0.001	0.57 (satisfactory)	0.48 (0.24–0.66)
MMSE + CDT ≤23 and ≤4	0.46 (0.36–0.51)	0.88 (0.75–0.96)	0.84 (0.66–0.95)	0.54 (0.46–0.59)	0.67 (0.55–0.79)	<0.001	0.39 (poor)	0.34 (0.11–0.47)

AUC, area under the receiver operating characteristic curve; CDT, Clock Drawing Test; CI, confidence interval; CUI, Clinical Utility Index; MMSE, Mini-Mental State Examination; NPV, negative predictive value; PPV, positive predictive value.

**Table 4:** Sensitivity and specificity and AUC of subtests of Cognistat (cognitive impairment 46 versus no cognitive impairment 33).

Subtest of Cognistat (test range)	Cutoff	Sensitivity	Specificity	AUC (95% confidence interval)	*P* value
Orientation (0–12)	≤8	0.09	1.00	0.64 (0.52–0.76)	0.036
Attention (0–8)	≤4	0.35	0.94	0.66 (0.54–0.78)	0.016
Comprehension (0–6)	≤4	0.09	1.00	0.68 (0.56–0.79)	0.008
Repetition (0–12)	≤9	0.15	1.00	0.61 (0.49–0.74)	0.088
Naming (0–8)	≤5	0.13	1.00	0.58 (0.46–0.71)	0.216
Construction (0–6)	≤2	0.39	0.97	0.73 (0.62–0.84)	0.001
Memory (0–12)	≤6	0.65	0.91	0.84 (0.75–0.93)	<0.001
Calculations (0–4)	≤2	0.13	1.00	0.59 (0.47–0.72)	0.170
Similarities (0–8)	≤3	0.15	1.00	0.66 (0.54–0.78)	0.018
Judgement (0–6)	≤3	0.24	0.94	0.64 (0.52–0.76)	0.038

AUC, area under the receiver operating characteristic curve.

## Appendix

### Questionnaire for Taking Part in the Study as a Cognitively Healthy Participant (Reference Person)

Please, answer by checking the yes or no square for each question.

1. Do you feel that your general memory works in a way that you can easily manage your everyday life?
  □ Yes □ No
2. Do you feel that your general thinking works in a way that you can easily manage your everyday life?
  □ Yes □ No
3. Do you feel that your general mood is such that it does not affect your everyday life negatively?
  □ Yes □ No
4. If you live with a partner or anyone else who knows you well, does he or she agree with your answers?
  □ Yes □ No

If the above questions are answered with YES, you can be considered for participation in the study in the reference group.

*Additional Questions *

1. Have you ever been diagnosed with cerebral infarction or haemorrhage (stroke) or some other types of ischemic brain dysfunction?
  □ Yes □ No
2. Have you ever been treated for a brain tumour?
  □ Yes □ No
3. Have you ever been treated for any brain infection?
  □ Yes □ No
4. Have you suffered any blows to the head in recent weeks?
  □ Yes □ No

If the above questions are answered with NO you can be considered for participation in the study in the reference group.
